# In silico investigation of cannabinoids from *Cannabis sativa* leaves as a potential anticancer drug to inhibit MAPK-ERK signaling pathway and EMT induction

**DOI:** 10.1007/s40203-024-00213-4

**Published:** 2024-05-06

**Authors:** Shabnoor Iqbal, Motlalepula Matsabisa

**Affiliations:** AMITD Department of Pharmacology, School of Clinical Medicine, Faculty of Health Sciences, University of the Free State, Bloemfontein, 9300 South Africa

**Keywords:** Cannabinoids, Cannabis, Cancer, Protein kinase, Mitogen-activated protein kinase, Epithelial to mesenchymal transition

## Abstract

Genes related to MAPK-ERK signaling pathways, and epithelial-mesenchymal transition induction is evolutionarily conserved and has crucial roles in the regulation of important cellular processes, including cell proliferation. In this study, six cannabinoids from *Cannabis sativa* were docked with MAPK-ERK signaling pathways to identify their possible binding interactions. The results showed that all the cannabinoids have good binding affinities with the target proteins. The best binding affinities were MEK- tetrahydrocannabinol (– 8.8 kcal/mol) and P13k-cannabinol (– 8.5 kcal/mol). The root mean square deviation was calculated and used two alternative variants (rmsd/ub and rmsd/lb) and the values of rmsd/lb fluctuated 8.6–2.0 Å and for rmsd/ub from 1.0 to 2.0 Å that suggests the cannabinoids and protein complex are accurate and cannot destroy on binding. The study analyzed the pharmacokinetic and drug-likeness properties of six cannabinoids from *C. sativa* leaves using the SwissADME web tool. Lipinski's rule of five was used to predict drug-likeness and showed that all compounds have not violated it and the total polar surface area of cannabinoids was also according to Lipinski's rule that is benchmarked of anticancer drugs. Cannabinoids are meet the requirements of leadlikeness and synthetic accessibility values showed they can be synthesized. The molecular weight, XLOGP3, solubility (log S), and flexibility (FLEX) are according to the bioavailability radar. The bioavailability score and consensus Log Po/w fall within the acceptable range for the suitable drug. Pharmacokinetics parameters showed that cannabinoids cannot cross the blood–brain barrier, have high GI absorption as well as cannabinoids are substrates of (CYP1A2, CYP2C19, CYP2C9, CYP2D6, and CYP3A4) but no substrate of P-glycoprotein. Based on these findings, the study suggests that cannabinoids are suitable drugs that could be used as effective inhibitors for target proteins involved in cancer pathways. Among the six cannabinoids, cannabinol and tetrahydrocannabinol exerted maximum binding affinities with proteins of MAPK-ERK signaling pathways, and their pharmacokinetics and drug-likeness-related profiles suggest that these cannabinoids could be superlative inhibitors in cancer treatment. Further in vitro, in vivo, and clinical studies are needed to explore their potential in cancer treatment.

**Supplementary Information:**

The online version contains supplementary material available at 10.1007/s40203-024-00213-4.

## Introduction

Numerous blockbuster medicines are produced, either directly or indirectly, from plants, which are the major source of novel pharmacologically active chemicals (Dehelean et al. 2021). Plants play a vital role in the treatment and prevention of diseases, even in conjunction with synthetic chemistry as a technique of drug discovery and production from plant scaffold molecules. Natural products will remain a vital source of therapeutic medicines. Many other natural products can be used as chemical models or as models for synthesis, and semi-synthesis of new molecules meant to cure human ailments, in addition to the ones that have been shown to have direct medicinal applications (Emhemmed et al. 2022).

The biological process known as the epithelial-to-mesenchymal transition (EMT) is important for several physiological and pathological circumstances, including cancer and tissue homeostasis. It converts epithelial cells into mesenchymal cells, which enhances their migratory and invasion potential while decreasing their ability to adhere and undergo apoptosis (Beach et al. 2011; Hamidi et al. 2022). While the EMT process increases cell motility and the production of mesenchymal markers (N-cadherin, fibronectin, and vimentin), it also attenuates cell–cell adhesion and downregulates epithelial indicators (E-cadherin) (Serrano-Gomez et al. 2016). Moreover, it is connected to drug treatment resistance, metastasis, and tumor growth. Through EMT, tumor cells at the primary tumor site can become migratory and invasive, which helps them spread to other organs and eventually metastasis (Pan et al. 2021; Huang et al. 2022). The signaling pathway RAF/MEK/ERK is responsible for controlling various cellular functions, such as cell division, proliferation, motility, and survival. In general, ERK1/2 activation stimulates cell proliferation, and many malignancies are characterized by its dysregulated activity (Sugiura et al. 2021). Additionally, the PI3K/Akt pathway plays a critical role in EMT by triggering downstream effectors that control cellular functions such as invasion, migration, and cell survival (Wei et al. 2019; Navaei et al. 2021). Furthermore, a variety of downstream targets, including transcription factors (FOXOs), cell cycle regulators (p21 and p27), and elements of the mTOR pathway (mTOR and p70S6K), are phosphorylated and regulated by active Akt kinase (Johnson et al. 2010; Farhan et al. 2017).

The usage of innovative psychoactive drugs that contain synthetic cannabinoids is on the rise (Castaneto et al. 2014). Products containing synthetic cannabinoids have effects resembling those of natural cannabis, but they are stronger, more hazardous, and have been linked to harmful side effects. A variety of psychotropic substances, primarily with high-potency cannabinoid receptor binding, are included in synthetic pharmaceuticals. The effects of natural cannabis and Δ9-tetrahydrocannabinol are mimicked by these synthetic drugs, but they cause more severe side effects, such as chest pain, tachycardia, anxiety, cognitive impairment, agitation, respiratory difficulties, muscle twitches, acute renal failure, and psychosis (Cohen and Weinstein 2018).

Numerous studies have been conducted on the application of cannabinoids as an anti-cancer treatment. It was found that it generally has beneficial and protective effects, preventing the growth and spread of tumors and reestablishing homeostasis. Therapeutic trials on the use of cannabinoids as an anti-cancer medication are currently being conducted, even though their therapeutic use in palliative care is well documented (Tomko et al. 2020).

It is anticipated that the pharmacokinetic and molecular docking data of cannabinoids and the proteins related to MAPK-ERK signaling pathways will help ensure that these drugs are successfully deciphered and developed into oncological healthcare since drug repurposing is a much faster and more cost-effective process than the de novo introduction of a new drug into the clinic.

## Materials and methods

### Ligand preparation

Through the PubChem database, we downloaded cannabinoids as SDF file and saved in “.pdbqt” format using BIOVIA/ Discovery Studio 2021 (Table 1).

**Table 1:** PubChem CIDs of ligands

Sr no	Ligand	PubChem CID
1.	Cannabigerol	5,315,659
2.	Cannabichromene	30,219
3.	Tetrahydrocannabivarin	93,147
4.	Cannabinol	2543
5.	Cannabidiol	644,019
6.	Tetrahydrocannabinol	16,078

### Optimization of the proteins

The 3D crystal structure of all the target proteins was downloaded as the Protein Data Bank (PDB) from https://www.rcsb.org/). Discovery Studio Visualizer 2021 was run to crystallize the target proteins with ligands. For this, all water molecules, small molecules, and ligands were deleted from the protein crystal structure and the optimized proteins were saved in pdbqt format (Table 2).

**Table 2:** PDB ID of selected proteins

Sr No	Protein	PDB ID
1	Vimentin	1gk4
2	Akt	1o6l
3	ERK1	2zoq
4	JNK	4yr8
5	mTOR	5flc
6	P13K	5itd
7	P38	5uoj
8	MEK	7juy
9	ERK2	2ERK
10	E-cadherin	4zt1

### Molecular docking analysis

The molecular docking analysis was performed on the PyRx Virtual screening tool (version 0.9) that assesses the suitable binding alignments of the ligands as well as the targeted proteins. The ligands are docked to the protein surface. Using this information, the optimal orientations of the ligand with the best binding affinities for the protein active sites were determined. Discovery Studio Visualizer 2021 was run to exhibit the ligands and protein binding with the respective amino acid residues. Based on the lowest binding affinities needed for the binding ligand to attach to the protein, the optimal poses were selected. The binding box of protein-ligands was built in Auto Dock software. The binding box-related files were analyzed in Auto Dock Vina built-in PyRx software, and PDBQT files were created.

### Homology modeling for Protein structure validation

Homology modeling was performed to validate the structure of the optimized protein data bank before molecular docking. A program called PROCHECK was used to validate modeled proteins. PROCHECK generates a Ramachandran plot and assesses the atomic distances, surface area, bond angle, and torsion angles (Vyas et al. 2012). The Ramachandran Plot was provided information on stable conformations of amino acid residues in term of phi (φ) and psi (ψ) angels as well as allowed and disallowed region for amino acid residues in high resolution, non-homologus protein crystal structures. Plot points represented the torsion angles of amino acid residues in a three-dimensional protein model.

### Pharmacokinetics and drug-likeness predictions

In computer-based drug development, pharmacokinetic, pharmacochemical, and drug-likeness studies have gained a lot of attention; they are used to determine the pharmacological structure by using the website (https://www.swissadme.ch). To create SMILES, the chemical structure of cannabinoids was drawn on Marvin and then immediately entered into the webpage to start the prediction process (Daina et al. 2017).

## Results and discussion

### Binding affinities of protein-ligands interactions

Molecular docking of cannabinoids with some of the proteins (ERK/MEK, and P13K/Akt/mTOR) of cancer pathways and EMT-related proteins (E-cadherin and vimentin) was determined using PyRx software. The maximum binding affinity was exerted by the tetrahydrocannabinol-MEK complex (− 0.8.8 kcal/mol), and the second highest binding affinity was observed in the cannabidiol-P13K complex (− 0.8.5 kcal/mol). The binding affinities were increased in order: cannabigerol < cannabidiol = cannabichromene < tetrahydrocannabivarin (Table 3). Few studies have evidently shown that cannabinoids interact with the MAPK-ERK -ERK signaling pathway and one earlier study depicted that cannabinoids interacted with and downregulated the MAPK-ERK signaling pathway to induce apoptosis in glioma cells (Ellert-Miklaszewska et al. 2005).

**Table 3:** Molecular docking results of cannabinoids-proteins of Pathways of MEK/ERK and P13K/Akt/mTOR, and proteins related to EMT induction

				Cannabigerol						
Protein	Vimentin	AKT	E-cadherin	mTOR	ERK2	ERK1	JNK	P13K	P38	MEK
Binding Affinities (kcal/mole)	– 7.3	– 6.6	– 5.4	– 6.7	– 6.5	– 7.1	– 6.7	– 6.9	– 5.9	– 6.3

				Cannabichromene						
Protein	Vimentin	AKT	E-cadherin	mTOR	ERK2	ERK1	JNK	P13K	P38	MEK
Binding Affinities (kcal/mole)	– 7.1	– 6.9	– 6.2	– 7.8	– 6.7	– 7.5	– 8	– 7.9	– 7.1	– 7.6

				Tetrahydrocannabivarin						
Protein	Vimentin	AKT	E-cadherin	mTOR	ERK2	ERK1	JNK	P13K	P38	MEK
Binding Affinities (kcal/mole)	– 7.6	– 6.6	– 6.4	– 8.3	– 7.6	– 7.6	– 7.9	– 7.8	– 7.4	– 7.8

				Cannabinol						
Protein	Vimentin	AKT	E-cadherin	mTOR	ERK2	ERK1	JNK	P13K	P38	MEK
Binding affinities (kcal/mole)	– 7.5	– 6.7	– 6.9	– 7.6	– 7.7	– 8.3	– 7.7	– 8.5	– 7.6	– 8

				Cannabidiol						
Protein	Vimentin	AKT	E-cadherin	mTOR	ERK2	ERK1	JNK	P13K	P38	MEK
Binding affinities (kcal/mole)	– 7.1	– 6.2	– 6.2	– 8.2	– 7.2	– 6.7	– 6.8	– 7.6	– 6.5	– 7.4

				Tetrahydrocannabinol						
Protein	Vimentin	AKT	E-cadherin	mTOR	ERK2	ERK1	JNK	P13K	P38	MEK
Binding affinities (kcal/mole)	– 7.9	– 6.7	– 6.5	– 8.3	– 7.3	– 8.6	– 7.9	– 7.8	– 7.5	– 8.8

### Protein–ligand interactions

**Fig. 1:**
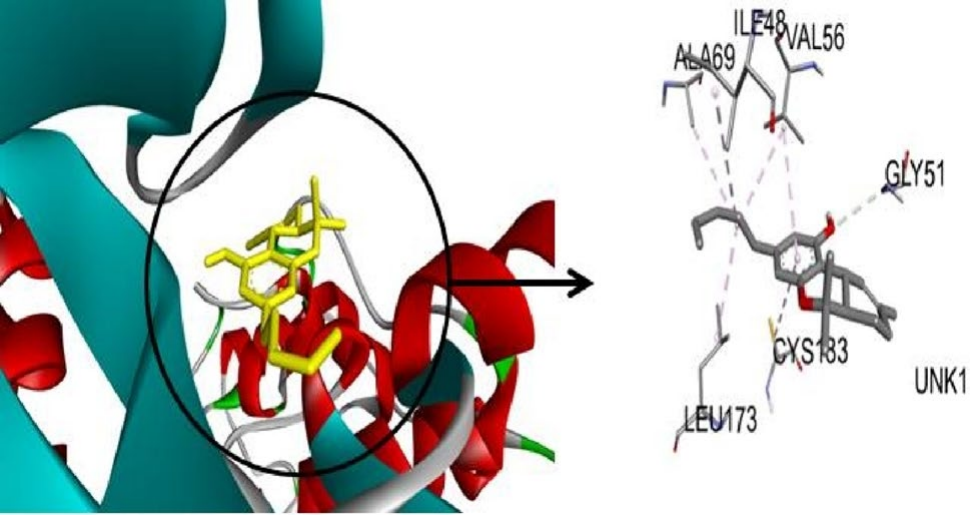
3 D visualization of ERK1-Tetrahydrocannabinol

**Fig. 2:**
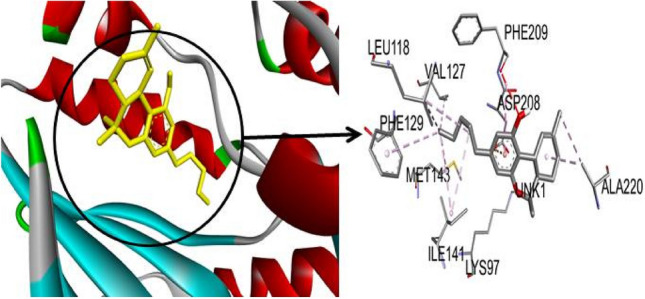
3D visualization of MEK-Tetrahydrocannabinol

The current study of *C. sativa* compounds provides promising information on the possible effectiveness of these phytochemicals against cancer. Protein–ligand interactions are crucial to drug development and offer an excellent understanding of the simulation. These protein–ligand interactions fall into four categories: ionic, hydrophobic, hydrogen bonds, and water bridges (ur Rashid et al. 2022). Hydrogen bonding, or H-bonds, is crucial for protein folding and interactions with ligands (Yunta 2017). Table 4 presents the binding interaction of cannabinoids (cannabidiol, tetrahydrocannabivarin, cannabigerol, cannabinol, cannabichromene, tetrahydrocannabinol) with proteins of the MAPK-ERK signaling pathway. ERK1- tetrahydrocannabinol complex was interacted by alkyl bonding with amino acid residues: Cys183, Ile48, Leu173, Val56, and Ala69 at the binding pocket (Fig. 1). Hydrogen bonding interactions were developed by MEK-tetrahydrocannabinol complex with amino acid residues of Lys97 and residues (Phe129, val127,leu118, Ile141, Met143, and Ala220) were developed alky bonding at the active site. Amongst the hydrophobic interactions, pi anion was developed between the MEK-tetrahydrocannabinol complex by Ala220 (Fig. 2). Hydrophobic and hydrogen interactions of tetrahydrocannabinol at binding sites of cannabinoid receptors were reported in the former study and developed interaction by Val, Phe, and Tyr residues at the active site of acetylcholinesterase receptor (Furqan et al. 2020; Aviz-Amador et al. 2021).

**Table 4:** Amino acid interactions of cannabinoids with target proteins at their active sites

				Cannabigerol						
Protein	Vimentin	Akt	E-cadherin	mTOR	ERK2	ERK1	JNK	P13K	P38	MEK
Alkyl Bond	ALA355, ALA35, MET347, PHE351, VAL389	PHE296, PHE163, PHE359, HIS355, PHE310	ILE4, LYS25,	–	LEU105, ALA50, VAL37	LEU360, LEU86, ARG87, PHE348, LEU352	LYS55, MET77, ILE86, VAL40, VAL158, MET108	LEU645, ARG683, VAL437	PRO6, PRO350, PHE8 ARG5	LYS737, ALA637, VAL670, CYS689, PHE688
H-Bond	TRY383	–	TRP2, GLU89,	UNK252	–	ARG189	SER155	ASP133, THR462	ASP88, PHE348	ASP750, ASN738
Pi-Anion bond	GLU382	–	–	–	–	–	–	GLU135	–	–
Pi-sigma bond	–	–	–	–	ILE29	–	LEU168			–
Pi- Sulphur Bond	–	–	MET92							–

				Cannabichromene						
Protein	Vimentin	Akt	E-cadherin	mTOR	ERK2	ERK1	JNK	P13K	P38	MEK
Alkyl Bond	PHE351, ALA355, VAL389, MET347, LEU387	PHE359, HIS355, PRO314, LYS191, ILE188, PHE163	ALA80, ILE4, MET92	–	LEU154, LEU105, ALA50, VAL37	LEU86, LEU360, LEU352, ARG87	LEU110, ILE32, ALA42, ALA53, LEU168, VAL40, VAL158, LYS55	VAL461, PRO466, TRP446	ILE346, VAL345	PRO124, LYS175, TYR125, LEU50, LEU54, PHE53
H-Bond	–	–	–	–	–	ILE190	MET111, GLU109	ARG683	–	ILE190
Pi-Anion bond	–	–	–	–	–	–	–	–	–	–
Pi-Cation bond	–	–	–	–	–	ARG189	–	–	–	
Pi-sigma bond	–	–	–	UNK1372	–	HIS195, PHE348	–	–	–	LEU42
Pi- Sulfur Bond	–	–	–	–		–	–	–	–	–

				Tetrahydrocannabivarin						
Protein	Vimentin	Akt	E-cadherin	mTOR	ERK2	ERK1	JNK	P13K	P38	MEK
Alkyl Bond	VAL389, MET347, PHE351, ALA355	PHE350, TYR351, LEU355	ASN168, ILE146	–	ALA50, LYS52, VAL37, LEU154, LEU105, ILE29	LEU86, LEU352	VAL40, LYS55, MET10, LEU11O,	PRO44, PRO447	TYR311	
H-Bond	–	–	–	–	MET106	–	–	–	ARG136	
Pi-Anion bond	–	–	–	–	–	–	–	–	–	
Pi-Cation bond	–	–	–	–	–	ARG18, ARG87	–	–	–	
Pi-sigma bond	–	–	–	UNK248, UNK120	–	–	VAL158	–	–	
Pi- Sulphur Bond	–	–	–	–	–	–	–	–	–	

				Cannabinol						
Protein	Vimentin	Akt	E-cadherin	mTOR	ERK2	ERK1	JNK	P13K	P38	MEK
Alkyl Bond	ARG378, TYR358	PHE350, LEU361,	ALA80, MET92, TYR36, ILE24, TRP2	–	LEU154, CYS164, ALA50, ILE82	LEU352, LEU86, ARG87, ILE190	MET149, ILE124, ALA91, LEU206	TYR836, ILE848, CYS838, ILE932, VAL850	ARG67, LEU171, LEU55	VAL627, PHE688, ALA637
H-Bond	–	PRO349	–	–	–	–	–	–	–	–
Pi-Anion bond	GLU375	–	–	–	–	–	–	–	–	–
Pi-Cation bond	–	–	–	–		ARG189	–	–	–	–
Pi-sigma bond	ILE362	TYR351	–	UNK538, UNK642	ILE29	PHE348	VAL196, VAL78	MET922	–	GLY620
Pi- Sulfur Bond	–	–	–	–	–	–	MET146	–	–	–

				Cannabidiol					–	
Protein	Vimentin	Akt	E-cadherin	mTOR	ERK2	ERK1	JNK	P13K	P38	MEK
Alkyl Bond	LEU168, VAL158, LEU110, ALA42	PHE163, LYS191, PHE310	ALA80, TYR36, MET92, ILE24	–	LYS52, VAL37, ALA33, ILE29, ALA50, LEU154	ALA349, LEU363, PHE346, PHE398	LEU168, VAL158, LEU110, ALA42	MET81, ILE633, ARG662	LEU167, LYS53	PHE740, PHE688, VAL627, ALA637, CYS689, VAL670, LYS737
H-Bond	ILE32	–	–	–	–	–	ILE32	–	–	–
Pi-Anion bond	–	–	–	–	LEU154	GLU367	–	–	–	–
Pi-Cation bond	–	–	–	–	–	–	–	–	–	–
Pi-sigma bond	–	–	–	UNK570	–	–	–	–	–	
Pi- Sulphur Bond	–	–	–	–	–	–	–	–	–	–

				Tetrahydrocannabinol						
Protein	Vimentin	Akt	E-cadherin	mTOR	ERK2	ERK1	JNK	P13K	P38	MEK
Alkyl Bond	LYS334, ILE362, TYR358	TYR351, ARG368	ALA145	–	LEU105, VAL37, ALA50, LYS52, CYS164	CYS183, ILE48, LEU173, VAL56, ALA69	MET108, VAL40, LYS55, LEU168	PRO449, PRO447	LYS165	PHE129, VAL127, LEU118, ILE141, MET143 ALA220
H-Bond	GLN359, GLN366	ARG347	ASN168	–	–	–	–	–	LEU164, GLU81, GLN133	LYS97
Pi-Anion bond	–		–	–	–	–	–	–	GLU163	ALA220
Pi-Cation bond	–	–	–	–	–	–	–	–	–	–
Pi-sigma bond	–	–	–	UNK120	ILE29	–	LEU168	–	–	–
Pi- Sulfur Bond	–	–	–	–	–	–	–	–	–	–

**Fig. 3:**
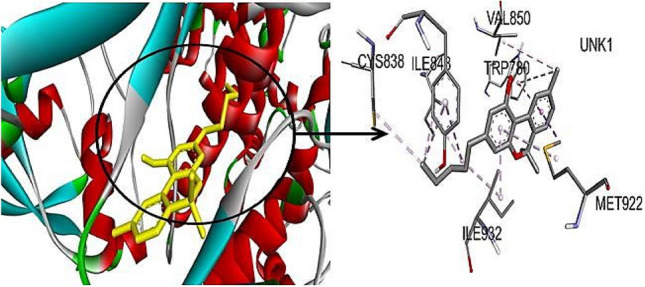
3D visualization of P13K-Cannabinol

**Fig. 4:**
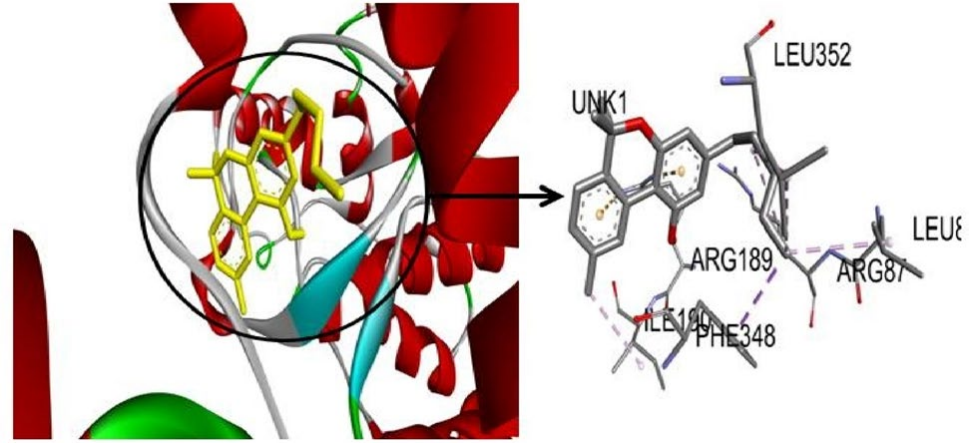
3D visualization of ERK1-Cannabinol

P13k-cannabinol was developed H-interaction with amino acid residues Try836, Ile848,cys838, Ile932, and val850 and interacted by covalent bonding (Pi sigma bond) with Met922 residue at binding pockets (Fig. 3). ERK1-cannabinol was developed noncovalent molecular interaction (Pi cation) by Arg189 residue and interacted by covalent bond (alkyl bond) by residues Leu352, Leu86, Arg8, Ile190 at the binding pockets (Fig. 4). Only Akt-cannabinol was found to be interacting via H-bond at the binding site by pro349 residue. An earlier study reported that cannabinol was developed hydrogen and alkyl bonding interaction with cannabinoid receptors (Aviz-Amador et al. 2021).

### Validation of protein–ligand complex

The protocol of docking was validated by RMSD values and calculated using the PyRx Virtual screening tool. The Root mean square deviation (RMSD) values were calculated by the mean distance between atoms of a position concerning the best fitting position are measured through only movable heavy atoms. The rmsd/lb (the RMSD lower bound) and rmsd/ub (the RMSD upper bound) are two different RMSD metrics that were provided. Two alternatives of RMSD metrics are given, rmsd/ub (RMSD upper bound)) and rmsd/lb ((RMSD lower bound), rmsd/ub matches every atom in one conformation with itself in the other conformation. The rmsd/ub values range from 8.6 to 2.0 Å, the computed rmsd/lb values fluctuate between 1.0 and 2.0 Å. These findings suggested that binding complex of cannabinoids (cannabidiol, cannabinol, cannabigerol, tetrahydrocannabinol, tetrahydrocannabivarin, and cannabichromene) with target proteins (Vimentin, mTOR, MEK, Akt, JNK, ERK1/2, P13K, P38, and E-cadherin), who gave the maximum binding energies was the accurate binding complex. The RMSD values suggested that cannabinoids and protein complexes are valid and accurate and did not face any damage after binding (Table 5). The present results of rmsd/lb also showed the accuracy of the docking methodology employed in this study by the fact that the RMSD value is less than the threshold value of 2.0 established to assess reliability (Benhander and Abdusalam 2022).

**Table 5:** Root mean square deviation (RMSD) values, which indicate the average distance between atoms at a point in relation to the best fitting position

PROTEIN–LIGAND	Distance from Best Mode	
	RMSD/Upper Bound	RMSD/Lower Bound
Vimentin-Tetrahydrocannabinol	3.828	1.582
Vimentin –cannabidiol	2.234	1.036
Vimentin –cannabichromene	5.461	1.897
Vimentin –cannabinol	6.997	1.777
Vimentin –cannabigerol	2.52	1.784
Vimentin –Terahydrocannabivarin	3.884	1.744
mTOR-Tetrahydrocannabinol	1.671	1.304
mTOR-cannabidiol	4.179	1.792
mTOR-cannabichromene	4.317	1.752
mTOR-cannabinol	6.886	1.937
mTOR-cannabigerol	2.339	1.255
mTOR-Terahydrocannabivarin	2.486	1.567
MEK-Tetrahydrocannabinol	3.28	1.43
MEK-cannabidiol	3.11	1.03
MEK-cannabichromene	4.55	1.89
MEK-cannabinol	5.027	1.729
MEK-cannabigerol	2.776	1.814
MEK-Terahydrocannabivarin	5.861	1.604
JNK-Tetrahydrocannabinol	2.098	1.733
JNK-cannabidiol	3.19	1.194
JNK-cannabichromene	3.468	1.435
JNK-cannabinol	3.468	1.435
JNK-cannabigerol	2.27	1.483
JNK-Terahydrocannabivarin	5.094	1.69
ERK2-Tetrahydrocannabinol	6.95	1.962
ERK2-cannabidiol	4.649	1.787
ERK2-cannabichromene	5.493	1.865
ERK2-cannabinol	3.724	1.557
ERK2-cannabigerol	2.964	1.256
ERK2-Terahydrocannabivarin	6.242	1.749
ERK1-Tetrahydrocannabinol	6.849	1.985
ERK1-cannabidiol	4.178	1.906
ERK1-cannabichromene	7.816	1.391
ERK1-cannabinol	6.576	1.198
ERK1-cannabigerol	8.642	1.59
ERK1-Terahydrocannabivarin	6.353	1.76
Akt-Tetrahydrocannabinol	6.888	1.36
Akt-cannabidiol	5.241	1.61
Akt-cannabichromene	8.272	1.57
Akt-cannabinol	6.495	1.43
Akt-cannabigerol	2.273	1.04
Akt-Terahydrocannabivarin	6.743	1.67
P13K-Tetrahydrocannabinol	4.833	1.39
P13K-cannabidiol	6.315	1.56
P13K-cannabichromene	7.269	1.39
P13K-cannabinol	7.644	1.88
P13K-cannabigerol	2.024	1.52
P13K-Terahydrocannabivarin	6.049	1.69
P38-Tetrahydrocannabinol	5.115	1.69
P38-cannabidiol	2.243	1.24
P38-cannabichromene	3.981	1.51
P38-cannabinol	3.802	1.59
P38-cannabigerol	5.155	1.95
P38-Terahydrocannabivarin	2.996	1.54
E-cadherin-Tetrahydrocannabinol	2.545	1.45
E-cadherin-cannabidiol	2.656	1.70
E-cadherin-cannabichromene	4.378	2.06
E-cadherin-cannabinol	2.379	1.31
E-cadherin-cannabigerol	3.975	1.52
E-cadherin-Tetrahydrocannabivarin	3.943	1.77

### Protein structure validation

Plots illustrate specific low energy conformations for ϕ (phi) and (psi) or stable conformations of amino acid residues along with favorable and unfavorable regions for amino acid residues in the plot. The result shows that the number of residues of protein in the allowed region is more than 80%. The number of residues is less than 1% in the disallowed region except ERK1 (1.4%). Some proteins such as E-cadherin, vimentin, Akt, JNK, and P38 have zero residues in the disallowed region (Table 6). This suggests that the optimized protein structure of MEK, ERK1/2/, P38, P13K, mTOR, Akt, E-cadherin, and vimentin are suitable for molecular docking and the respective ligands can interact at their binding pockets with stable binding. The majority of points of the Ramachandran plots are situated in favorable regions, suggesting that the majority of the dihedral angles of amino acid residues are in appropriate ranges that assisted to develop stable protein–ligand complex (Hollingsworth and Karplus 2010).

**Table 6:**
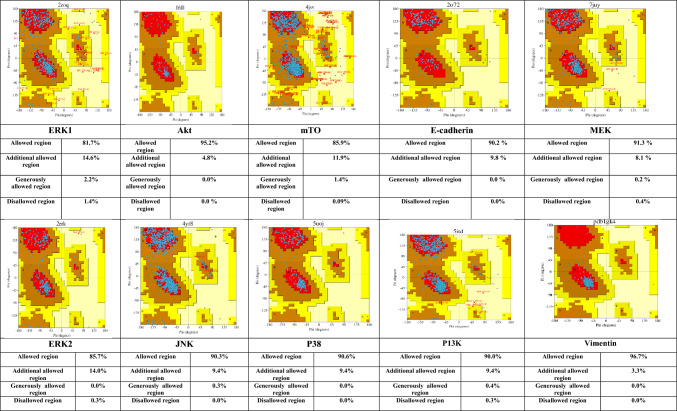
Ramachandran plot of each protein shows the overview of allowed and disallowed regions of torsion angle values,

The regions in the red indicates favoured region, yellow for allowed region, light yellow for generously allowed region of amino ac

### Drug-likeness predictions and pharmacokinetics

The in silico pharmacokinetic and drug-likeness properties of the six cannabinoids from the leaves of *C.* *sativa* are reported (Table 7) and the values were predicted by the SwissADME. Lipinski's rule of five, which states that if any small molecule violates more than two of these criteria (molecular weight ≤ 500 g/mol, number of hydrogen bond donors ≤ 5, number of hydrogen bond acceptors ≤ 10, calculated logP ≤ 5), the molecule is said to be impermeable or badly absorbed (Lipinski et al. 1997). This rule was used to predict the drug-likeness of the cannabinoid compounds (Table 7). The cannabinoids have 1 violation (MLOGP > 4.15) of the Lipinski rules of five except tetrahydrocannabivarin (0 violation).All cannabinoids have a good bioavailability score (0.55) that verifies their drug-likeness properties of cannabinoids (Ibrahim et al. 2021). Six cannabinoids meet the requirements for leadlikeness that is, a molecular entity that can be optimized (Teague et al. 1999). However, the synthetic accessibility value of these cannabinoids is less than 6, which suggests their possibility to be synthesized. The primary concept of the SwissADME Synthetic Accessibility (SA) Score is that synthesis ease is correlated with the frequency of molecular fragments in "really" attainable compounds and the score ranges from 1 (very easy to synthesize) to 10 (very hard to synthesize) (Ertl and Schuffenhauer 2009). Furthermore, only tetrahydrocannabivarin has less than 5 values of Consensus Log Po/w which is an average of the five lipophilicity predictions, falling within an acceptable range (Alminderej et al. 2020)**.** According to Lipinski’s rule of five, the range of total polar surface area (TPSA) should be of 0–140 that is benchmark for anticancer drugs and cannabinoids are compliance with Lipinski’s rule of five**.** The TPSA of all the compounds was between 29.46 and 40.46 Å^2^ (Jagannathan 2019). Cannabigerol, cannabichromene, tetrahydrocannabivarin, cannabinol, cannabidiol, and tetrahydrocannabinol have numerous rotatable bonds, which are less than 10. This indicates that these compounds have stable conformation and bioavailable if consumed orally (Rai et al. 2023).

**Table 7:** Physicochemical parameters of cannabinoids leaves of *Cannabis sativa* using SwissADME

Properties	Cannabigerol	Cannabichromene	Tetrahydrocannabivarin	Cannabinol	Cannabidiol	Tetrahydrocannabinol
Physiochemical						
Molecular weight	316.48 g/mol	314.46 g/mol	286.41 g/mol	310.43 g/mol	314.46 g/mol	314.46 g/mol
Num. of rotatable bonds	9	7	2	4	6	4
Num. H-bond acceptors	2	2	2	2	2	2
Num. H-bond donors	2	1	1	1	2	1
Consensus Log Po/w	5.74	5.74	4.68	5.21	5.2	5.33
TPSA	40.46 Å^2^	29.46 Å^2^	29.46 Å^2^	29.46 Å^2^	40.46 Å^2^	29.46 Å^2^
Drug Likeness	Yes; 1 violation: MLOGP > 4.15	Yes; 1 violation: MLOGP > 4.15	Yes; 0 violation	Yes; 1 violation: MLOGP > 4.15	Yes; 1 violation: MLOGP > 4.15	Yes; 1 violation: MLOGP > 4.15
Lipinski						
Bioavailability Score	0.55	0.55	0.55	0.55	0.55	0.55
Medicinal Chemistry	No; 2 violations: Rotors > 7, XLOGP3 > 3.5	No; 1 violation: XLOGP3 > 3.5	No; 1 violation: XLOGP3 > 3.5	No; 1 violation: XLOGP3 > 3.5	No; 1 violation: XLOGP3 > 3.5	No; 1 violation: XLOGP3 > 3.5
Lead Likeness						
Synthetic accessibility	3.14	4.26	4.05	3.39	4.05	4.27

The current state of drug development is characterized by pharmacokinetic and safety profiles of novel chemical entities (NCE) (Chen et al. 2018). In the early stages of drug development, several computational techniques could assist us in making predictions about the drug-likeness activity and possible toxicity of novel molecules. The ADMESwiss predicted that all six cannabinoid compounds would be highly absorbed via the intestine.

Predictions were made for the pharmacokinetic characteristics of absorption, distribution, skin penetration, metabolism, biotransformation, and excretion. This prediction tool suggests that tetrahydrocannabinol, tetrahydrocannabivarin, cannabidiol, and tetrahydrocannabinol except cannabichromene and cannabigerol can cross the blood–brain barrier. This prediction makes it clear that every component had high GI absorption, as seen in Table 7. The chemicals that are blood–brain permeant may, upon metabolism, produce toxicants that are damaging to the brain and bloodstream. Potential medications for transdermal and oral delivery can be identified and predicted using the skin permeability model. Cannabigerol was discovered to be the cannabinoid more skin permeant. According to the model, a molecule is considered less skin permeant if its log Kp value is more negative which supports the present findings (Daina et al. 2017).

The potential for cannabinoids to function as either a substrate or an inhibitor of P-gp was assessed; the findings showed that cannabinoids are not substrates of P-gp. An earlier study reported if there is no P-glycoprotein (P-gp) substrate that suggests the compound has high bioavailability and intestinal absorption, this supports present findings (Montanari and Ecker 2015). Prediction reveals that cannabinoids are substrate of CYP1A2 (except cannabigerol), CYP2C19 (except cannabigerol and cannabidiol), CYP2C9 (except cannabichromene and cannabidiol), CYP2D6, and CYP3A4 (Table 8). Previous Cytochrome P450 (CYP) enzymes play a crucial role in drug removal through metabolic transformation, making molecule-enzyme interactions critical (Daina et al. 2017). Because inhibition of these isoenzymes reduces the solubility and accumulation of the drug or its metabolites, it may have unintended negative side effects (Mishra and Dahima 2019).

**Table 8:** Pharmacokinetics parameters of cannabinoids leaves of *Cannabis sativa* using by SwissADME

Parameters of pharmacokinetics	Tetrahydrocannabivarin	Cannabigerol	Cannabinol	Tetrahydrocannabinol	Cannabichromene	Canabidiol
GI absorption	High	High	High	High	High	High
BBB permeant	Yes	No	Yes	Yes	No	Yes
P-gp substrate	No	No	No	No	No	No
CYP1A2 inhibitor	No	Yes	No	No	No	No
CYP2C19 inhibitor	No	Yes	No	No	No	Yes
CYP2C9 inhibitor	No	No	No	No	Yes	Yes
CYP2D6 inhibitor	No	No	No	No	No	No
CYP3A4 inhibitor	No	No	No	No	No	No
Log Kp (skin permeation)	– 3.87 cm/s	– 2.96 cm/s	– 3.86 cm/s	– 3.27 cm/s	– 3.35 cm/s	– 3.59 cm/s

**Fig. 5:**
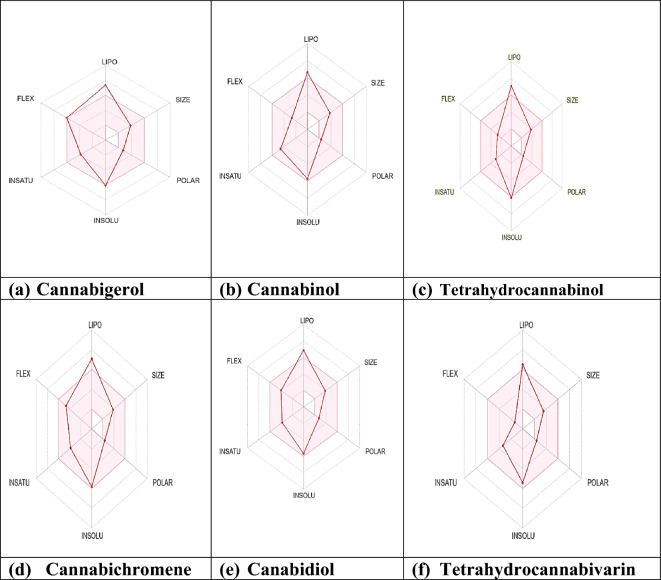
Bioavailability radars (**a**–**f**)

All characteristics, including XLOGP3 (-0.7 to + 5.0), Molecular weight (150 to 500 g/mol), Solubility (log S not exceeding 6), and Flexibility (FLEX) (not exceeding 9 rotatable bonds), are within the permissible range, according to the bioavailability radar (Fig. 5). The present finding fall within the standard drug flexibility and solubility criteria according to that Log S for solubility should not be greater than 6. XLOGP3 for lipophilicity should fall between -0.7 to + 6.0. The molecule should have no more than 9 rotatable bonds for flexibility (Cheng et al. 2012). The drug-likeness and drug score of cannabinoids suggest that they are better suited for usage as medicines.

## Conclusion

This study docked six cannabinoids from *Cannabis sativa* with proteins related to MAPK-ERK signaling pathways and proteins related to epithelial-mesenchymal transition (EMT) induction. The pharmacokinetics and drug-likeness properties of six cannabinoids (tetrahydrocannabivarin, cannabigerol, cannabinol, cannabidiol, tetrahydrocannabinol, and cannabichromene) from *C. sativa* leaves were also analyzed. All the cannabinoids had expressed good binding affinities and their drug-likeness as well as pharmacokinetics elucidated that they may be used as active drugs or inhibitors to downregulate Akt, mTOR, JNK, MEK, P38, P13K, ERK1/2, vimentin, E-cadherin. According to drug-likeness, pharmacokinetic, and binding affinities, out of the six cannabinoids,. tetrahydrocannabinol and cannabinol may be the best inhibitors of proteins related to the MAPK-ERK signaling pathway and EMT induction. This shows that cannabinoids may be used to formulate excellent anticancer medications. The study suggests that cannabinoids are better suited as drugs and to acquire a better knowledge of these medications, improve safe use, and effective prescribing, more clinical research in the actual patient groups for whom prescribing may be considered is required.

### Supplementary Information

Below is the link to the electronic supplementary material.

Supplementary file1 (DOCX 3683 KB)

## Data Availability

Data is available in manuscript as well as supplementary file and any additional data will be available on request.
