# Supplementary material for: In silico investigation of cannabinoids from Cannabis sativa leaves as a potential anticancer drug to inhibit MAPK-ERK signaling pathway and EMT induction
Source: In Silico Pharmacol. 2024 May 6;12(1):41. doi: 10.1007/s40203-024-00213-4 (PMC11074091; doi:10.1007/s40203-024-00213-4)
Supplement: Supplementary file 1 — Supplementary file1 (DOCX 3683 KB) [file 40203_2024_213_MOESM1_ESM.docx]

Table S 1. 3 D Structure of Cannabinoids and proteins related to MEK/AKT/ERK1/2/P13K/Vimentin/E-cadherin signaling pathways

| **3 D Structure of Tetrahydrocannabivarin-vimentin interaction** | **3 D Structure of Tetrahydrocannabivarin-E-cadherin interaction** | **3 D Structure of Tetrahydrocannabivarin-AKT interaction** | **3 D Structure of Tetrahydrocannabivarin-MEK interaction** | **3 D Structure of Tetrahydrocannabivarin-mTOR interaction** |
| --- | --- | --- | --- | --- |
| **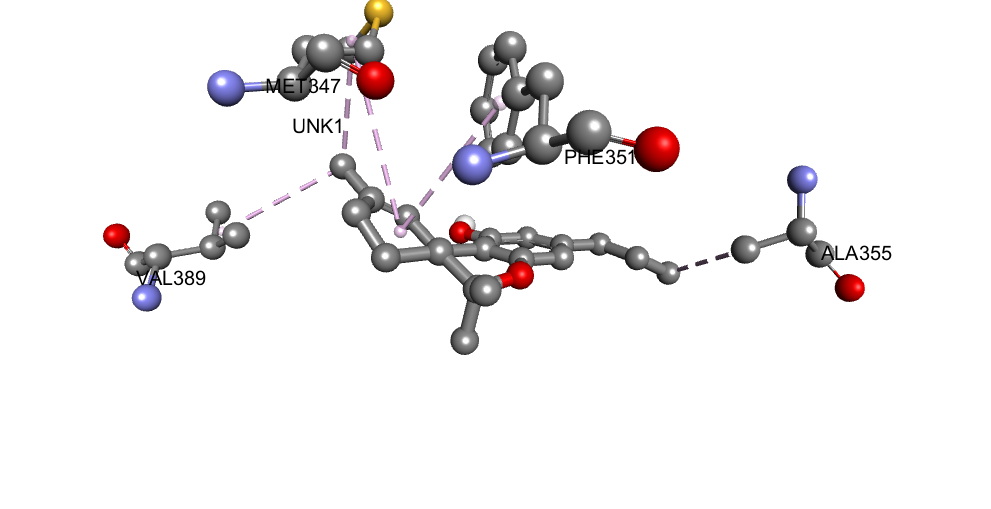** | **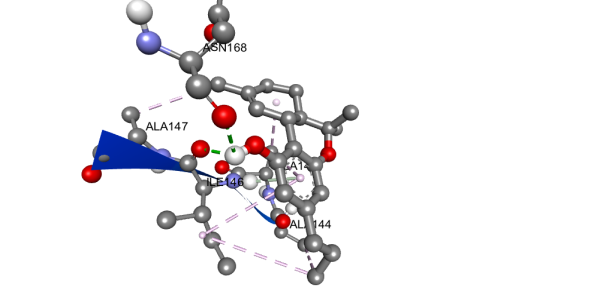** | **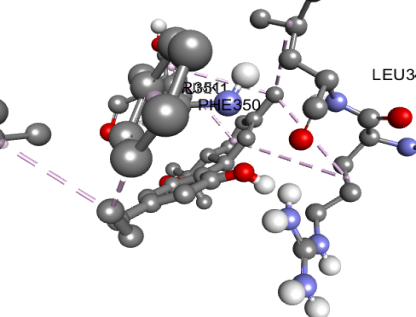** | **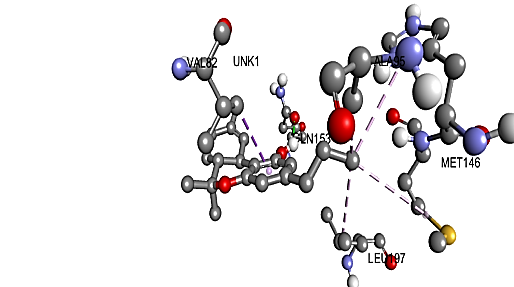** | **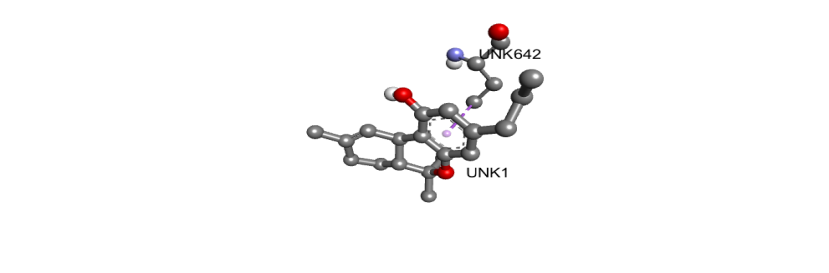** |
| **3 D Structure of Tetrahydrocannabivarin-ERK 1 interaction** | **3 D Structure of Tetrahydrocannabivarin-ERK 2 interaction** | **3 D Structure of Tetrahydrocannabivarin-JNK interaction** | **3 D Structure of Tetrahydrocannabivarin-P13K interaction** | **3 D Structure of Tetrahydrocannabivarin-P38 interaction** |
| **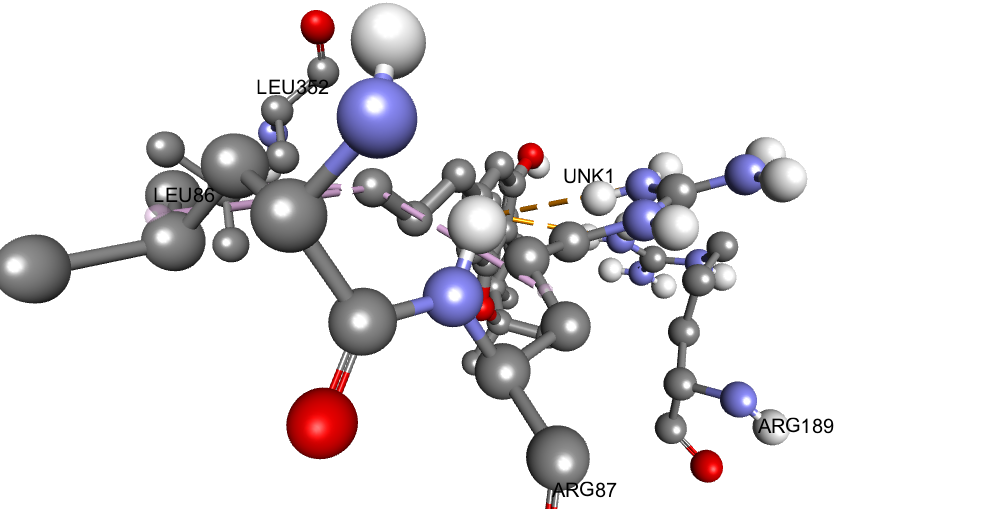** | **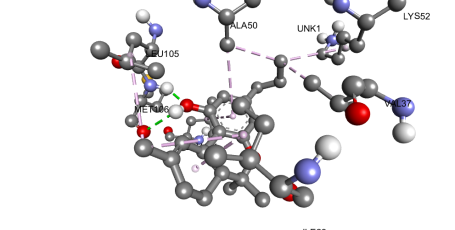** | **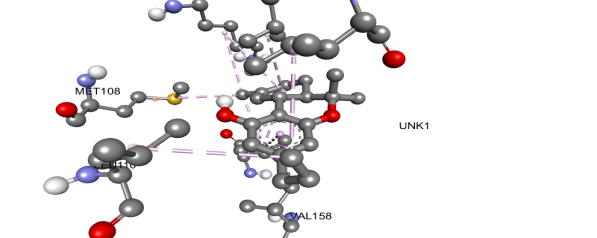** | **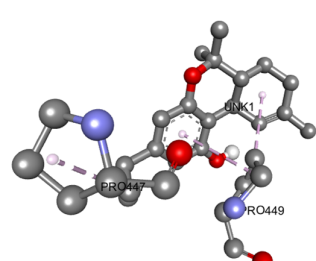** | **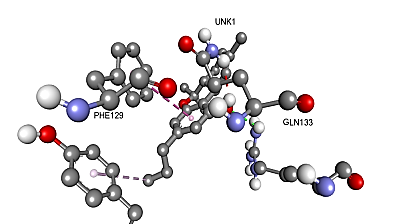** |

| **3 D Structure of Canabigerol vimentin interaction** | **3 D Structure of Cannabigerol-E-cadherin interaction** | **3 D Structure of Cannabigerol -AKT interaction** | **3 D Structure of Cannabigerol -MEK interaction** | **3 D Structure of Cannabigerol -mTOR interaction** |
| --- | --- | --- | --- | --- |
| 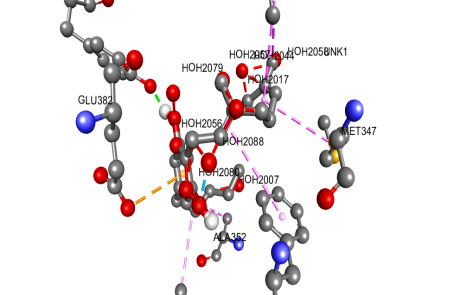 | 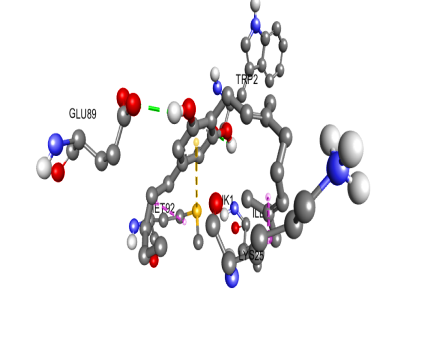 | 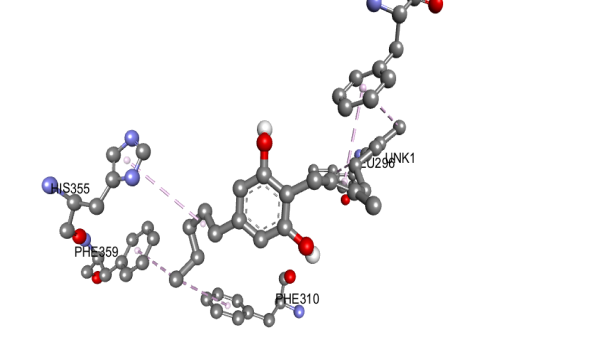 | 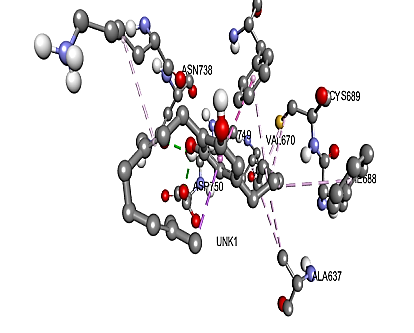 | 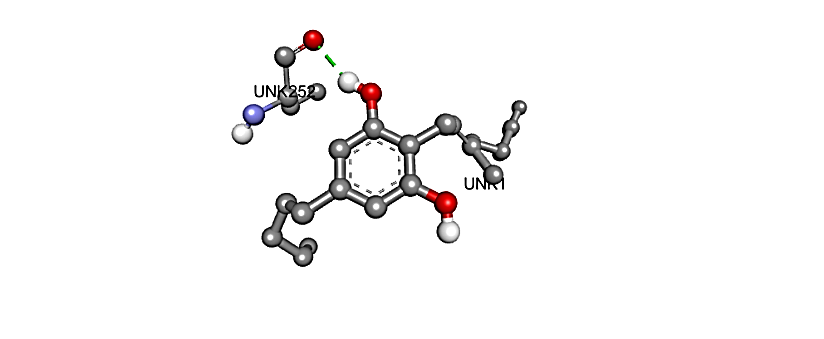 |
| **3 D Structure of Canabigerol -ERK 1 interaction** | **3 D Structure of Canabigerol -ERK 2 interaction** | **3 D Structure of Canabigerol -JNK interaction** | **3D Structure of Canabigerol -P13K interaction** | **3 D Structure of Canabigerol -P38 interaction** |
| 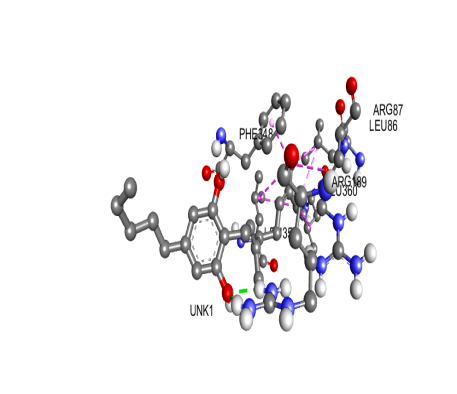 | 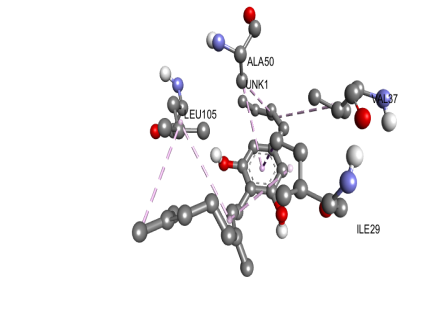 | 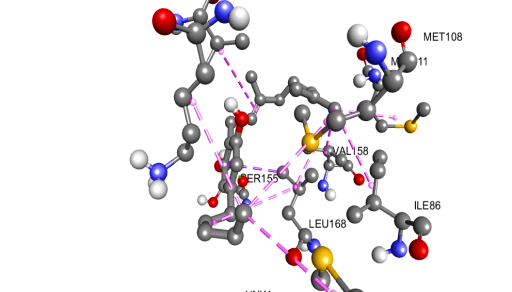  , | 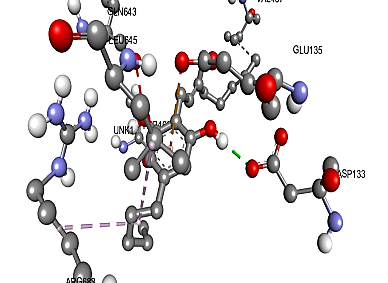 | 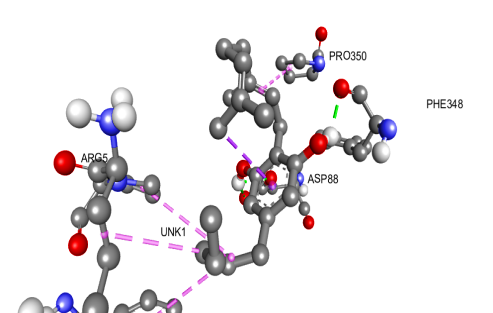 |
| **3 D Structure of Cannabidiol -vimentin interaction** | **3 D Structure of Cannabidiol-E-cadherin interaction** | **3 D Structure of cannabidiol-AKT interaction** | **3 D Structure of cannabidiol -MEK interaction** | **3 D Structure of cannabidiol -mTOR interaction** |
| 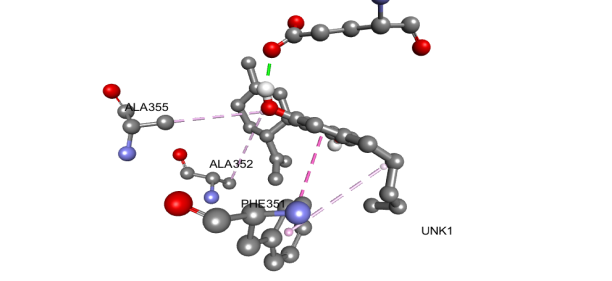 | 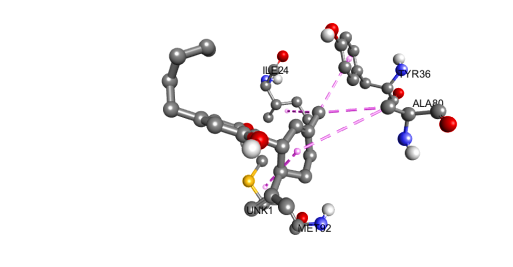 | 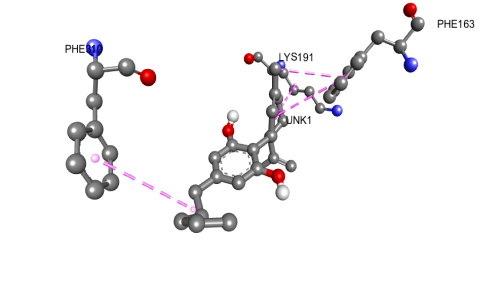 | 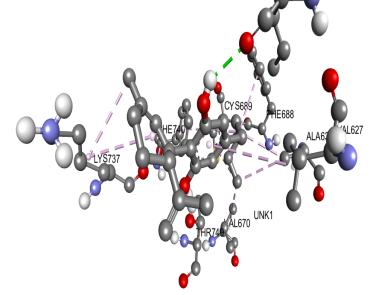 | 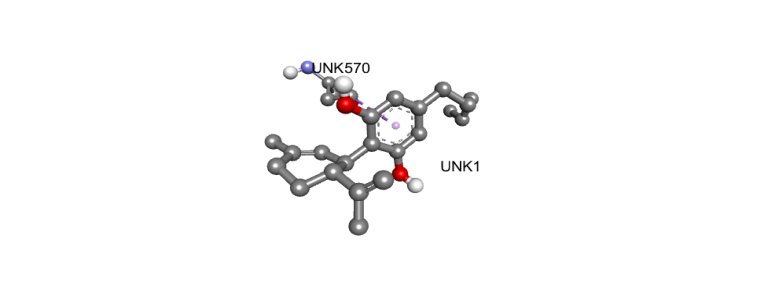 |
| **3 D Structure of Cannabidiol -ERK 1 interaction** | **3 D Structure of Cannabidiol -ERK 2 interaction** | **3 D Structure of Cannabidiol -JNK interaction** | **3 D Structure of Cannabidiol -P13K interaction** | **3 D Structure of Cannabidiol -P38 interaction** |
| 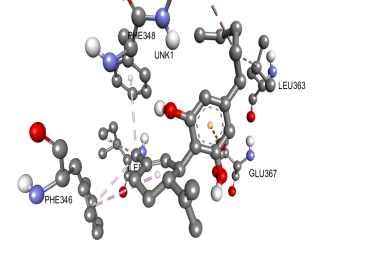 | 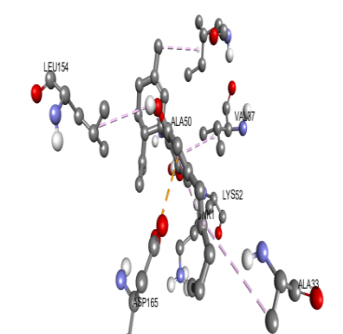 | 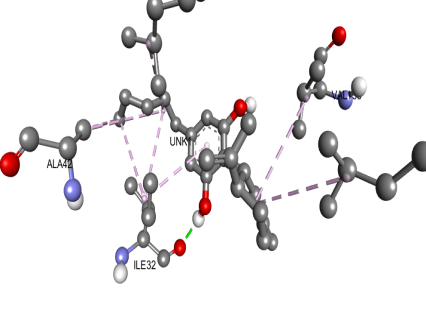 | 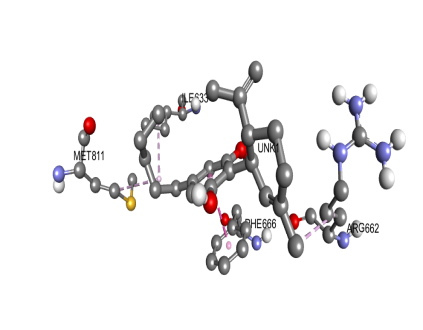 | 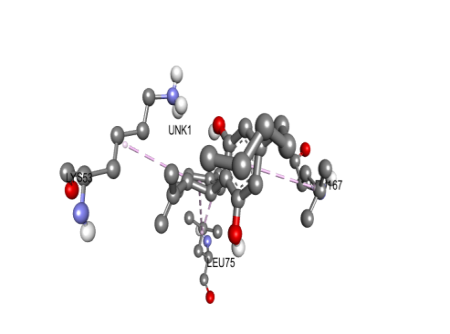 |

| **3 D Structure of Cannabichromene-vimentin interaction** | **3 D Structure of Cannabichromene -E-cadherin interaction** | **3 D Structure of Cannabichromene -AKT interaction** | **3 D Structure of Cannabichromene -MEK interaction** | **3 D Structure of Cannabichromene -mTOR interaction** |
| --- | --- | --- | --- | --- |
| *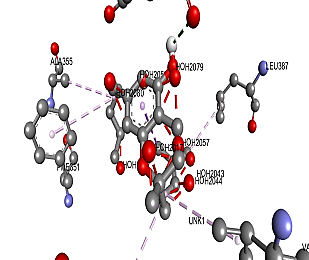* | 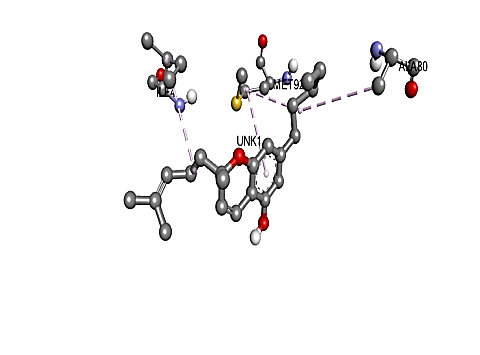 | 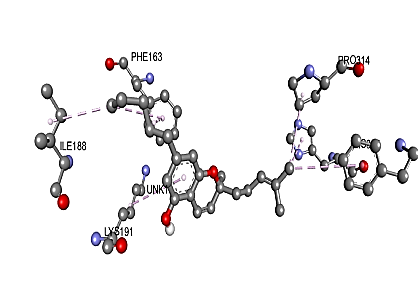 | 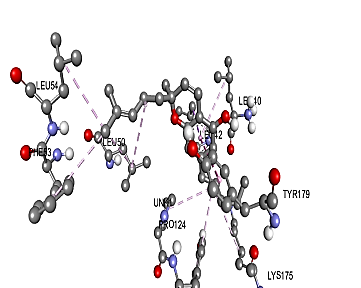 | 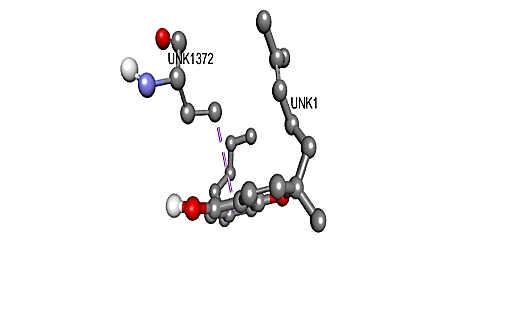 |
| **3 D Structure of Cannabichromene -ERK 1 interaction** | **3 D Structure of Cannabichromene -ERK 2 interaction** | **3 D Structure of Cannabichromene -JNK interaction** | **3 D Structure of Cannabichromene -P13K interaction** | **3 D Structure of Cannabichromene -P38 interaction** |
| 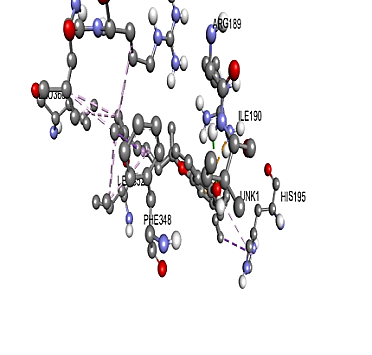 | 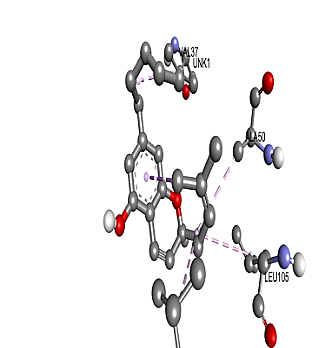 | 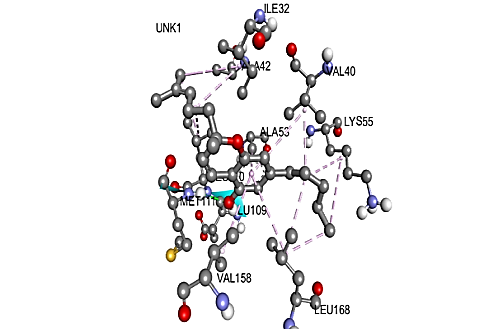 | 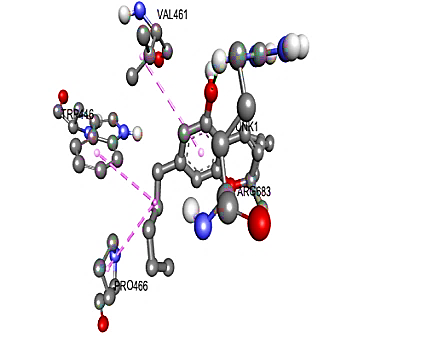 | 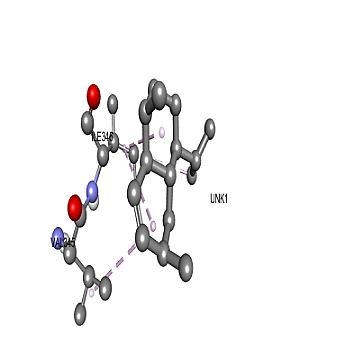 |

| **3 D Structure of Tetrahydrocannabinol-vimentin interaction** | **3 D Structure of Tetrahydrocannabinol -E-cadherin interaction** | **3 D Structure of Tetrahydrocannabinol -AKT interaction** | **3 D Structure of Tetrahydrocannabinol -MEK interaction** | **3 D Structure of Tetrahydrocannabinol -mTOR interaction** |
| --- | --- | --- | --- | --- |
| 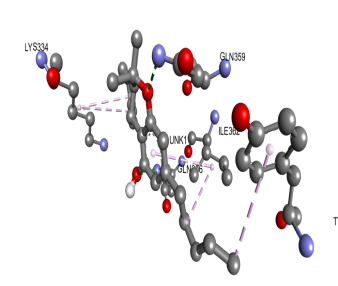 | 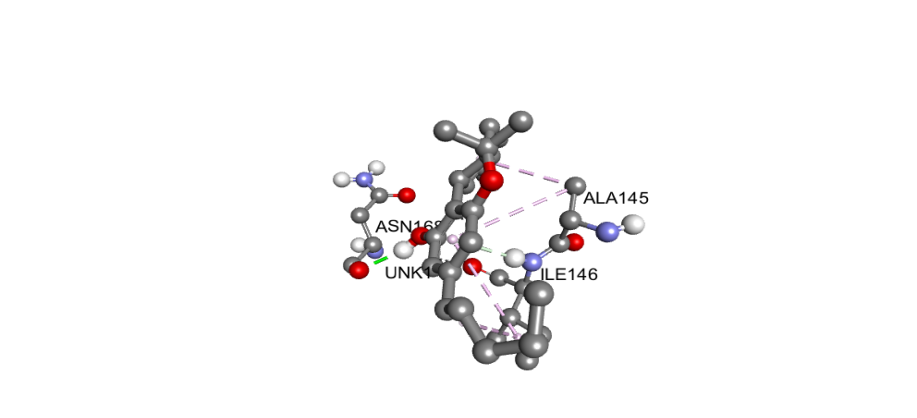 | 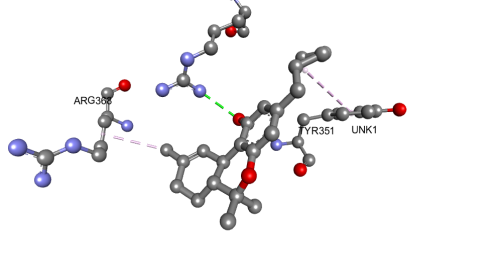 | 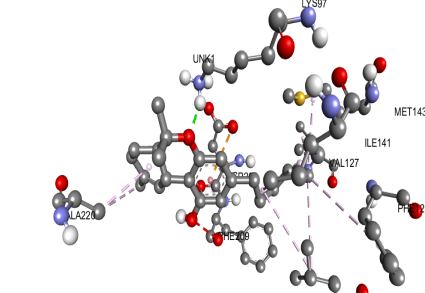 | 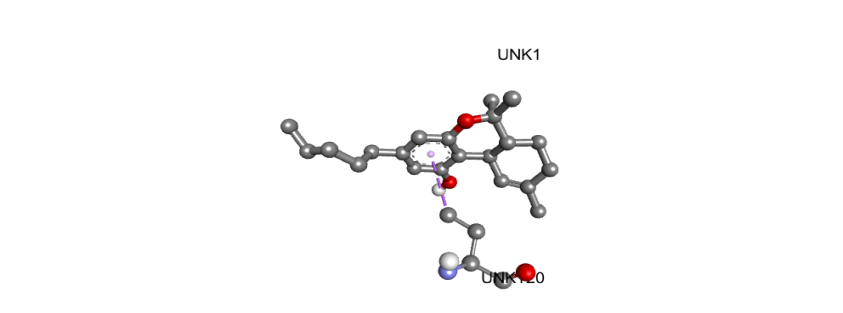 |
| **3 D Structure of Tetrahydrocannabinol -ERK 1 interaction** | **3 D Structure of Tetrahydrocannabinol -ERK 2 interaction** | **3 D Structure of Tetrahydrocannabinol -JNK interaction** | **3 D Structure of Tetrahydrocannabinol -P13K interaction** | **3 D Structure of Tetrahydrocannabinol -P38 interaction** |
| 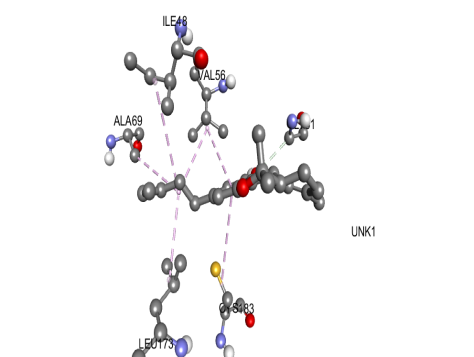 | 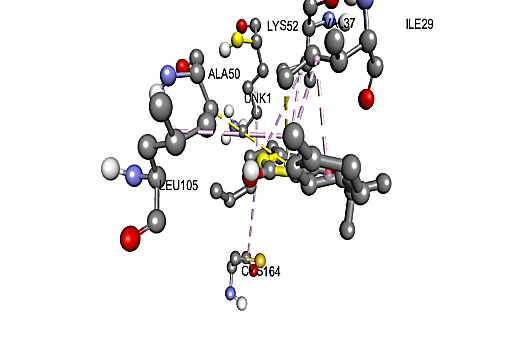 | 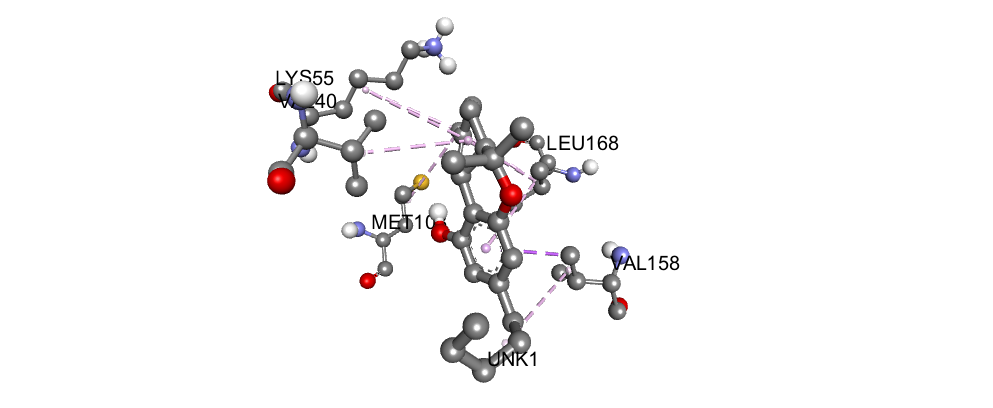 | 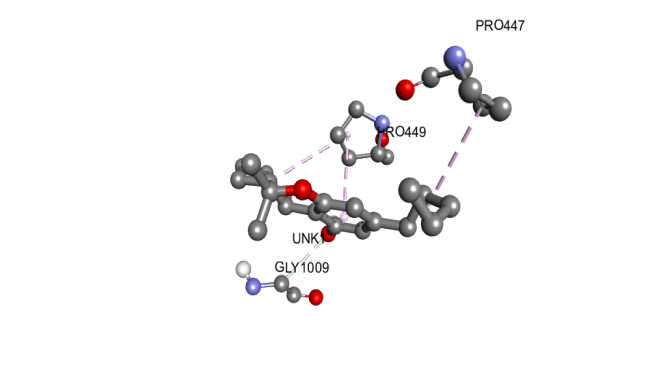 | 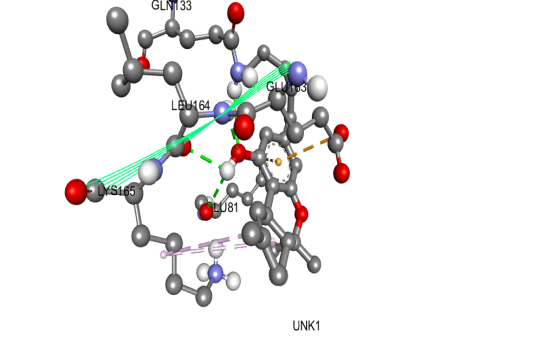 |

| **3 D Structure of Cannabinol -vimentin interaction** | **3 D Structure of Cannabinol -E-cadherin interaction** | **3 D Structure of Cannabinol -AKT interaction** | **3 D Structure of Cannabinol -MEK interaction** | **3 D Structure of Cannabinol -mTOR interaction** |
| --- | --- | --- | --- | --- |
| 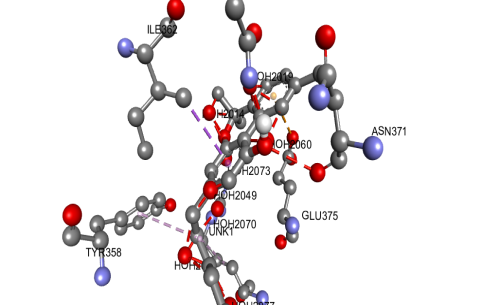 | 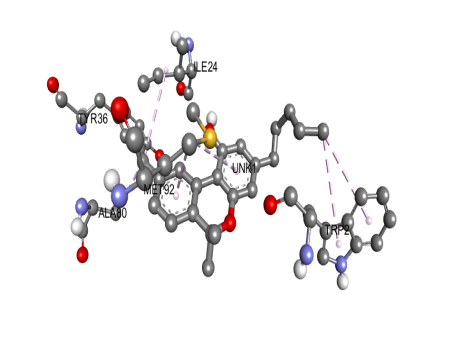 | 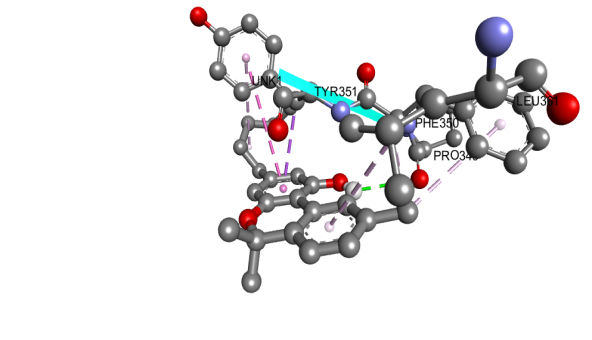 | 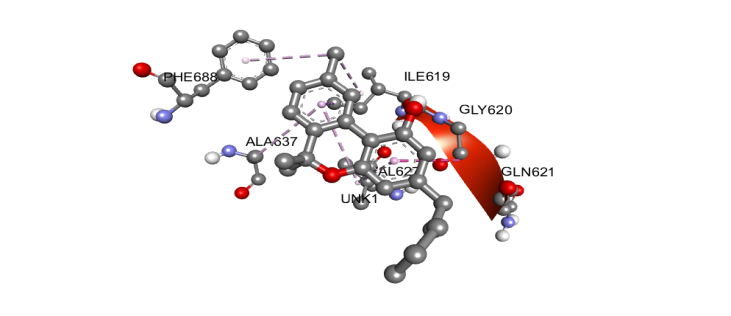 | 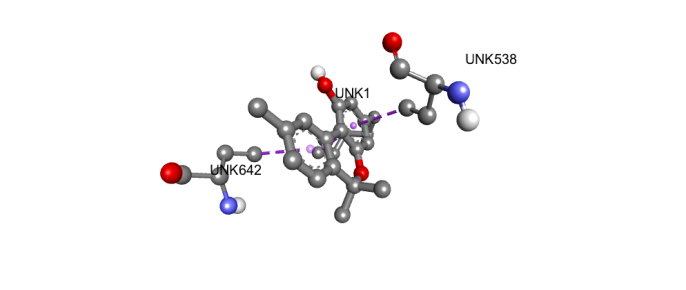 |
| **3 D Structure of Cannabinol -ERK 1 interaction** | **3 D Structure of Cannabinol -ERK 2 interaction** | **3D Structure of Cannabinol -JNK interaction** | **3 D Structure of Tetrahydrocannabivarin-P13K interaction** | **3 D Structure of Tetrahydrocannabivarin-P38 interaction** |
| 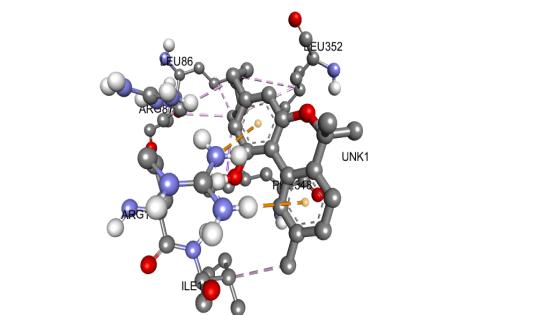 | 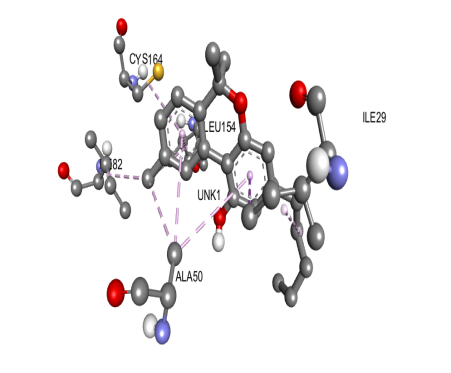 | 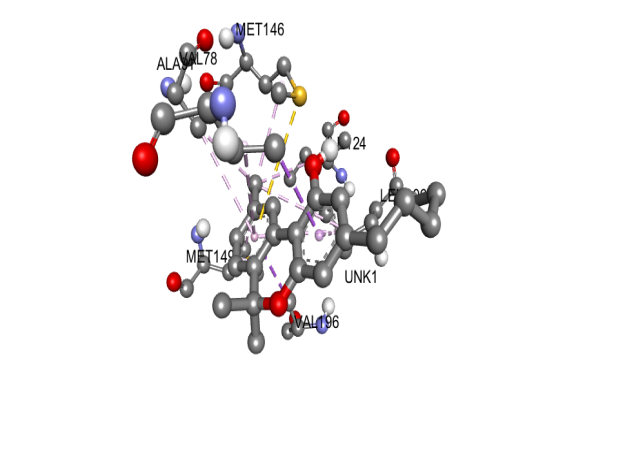 | 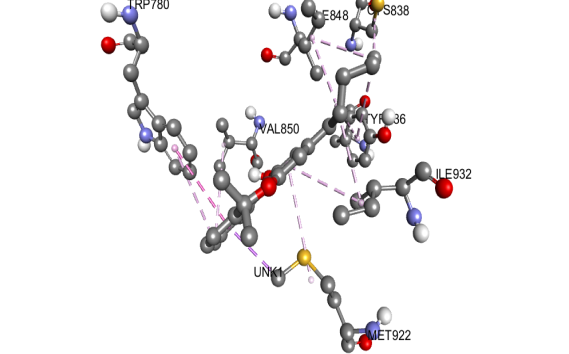 | 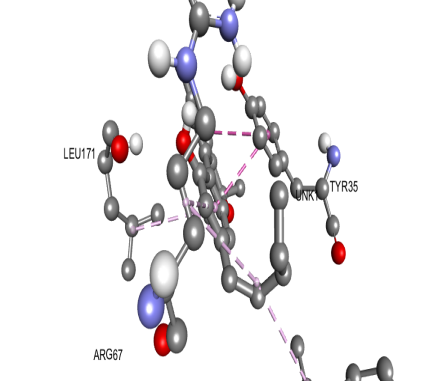**\** |
